# Supplementary material for: Staphylococcus aureus Genomic Analysis and Outcomes in Patients with Bone and Joint Infections: A Systematic Review
Source: Int J Mol Sci. 2023 Feb 6;24(4):3234. doi: 10.3390/ijms24043234 (PMC9967247; doi:10.3390/ijms24043234)
Supplement: Supplementary file 1 [file ijms-24-03234-s001.zip › ijms-2188389-supplementary.pdf]

Review

Staphylococcus aureus genomic analysis and outcome in patients with bone and joint infections: a systematic review

Supplementary materials 1) Search strategy

(("Staphylococcus aureus "[TW] OR "MRSA"[TW] OR "MSSA"[TW] OR "S. aureus"[TW])
AND
("osteomyelitis"[TW] OR "bone and joint infections"[TW] OR "implant-related infections"[TW] OR "periprosthetic joint infections"[TW] OR "Prosthetic joint infections"[TW] OR "bone infection"[TW] OR "joint infection"[TW] OR "osteoarticular infections"[TW] OR "orthopedic infections"[TW] OR "Septic arthritis"[TW] OR osteomyelitis[MH] OR joint prosthesis implantation [MH] OR Arthritis, Infectious[MH]) **AND**
("genotype"[TW] OR "genotypic"[TW] OR "genome"[TW] OR "genotyping"[TW] OR "gene"[TW] OR "genomic"[TW] OR " Pulsed-Field Gel Electrophoresis"[TW] OR "PFGE"[TW] OR "pulsotype"[TW] OR "MLST"[TW] OR "sequence type"[TW] OR "multi-locus sequence types"[TW] OR "clonal complex"[TW] OR "clonal lineage"[TW] OR "PCR"[TW] OR "Polymerase chain reaction"[TW] OR "microarray"[TW] OR "whole-genome sequencing"[TW] OR "virulence gene"[TW] OR "virulence factor"[TW] OR "adhesins"[TW] OR "toxins"[TW] OR whole genome association analysis [MH] OR genomics[MH] OR genome[MH] OR virulence factor[MH])
AND
(2000/01/01:3000[dp])) NOT ("animals"[MH] NOT "humans"[MH]) NOT (Mice[MeSH] OR "Models, Animal"[MH])
October, 11th, 2022

Supplementary materials 2) References of studies included in the review

1. Trouillet-Assant, S.; Lelièvre, L.; Martins-Simões, P.; Gonzaga, L.; Tasse, J.; Valour, F.; Rasigade, J.-P.; Vandenesch, F.; Muniz Guedes, R.L.; Ribeiro de Vasconcelos, A.T.; et al. Adaptive Processes of *Staphylococcus aureus* Isolates during the Progression from Acute to Chronic Bone and Joint Infections in Patients. *Cell Microbiol* **2016**, *18*, 1405–1414, doi:10.1111/cmi.12582.
2. Kok, E.Y.; Vallejo, J.G.; Sommer, L.M.; Rosas, L.; Kaplan, S.L.; Hulten, K.G.; McNeil, J.C. Association of Vancomycin MIC and Molecular Characteristics with Clinical Outcomes in Methicillin-Susceptible *Staphylococcus aureus* Acute Hematogenous Osteoarticular Infections in Children. *Antimicrob Agents Chemother* **2018**, *62*, e00084-18, doi:10.1128/AAC.00084-18.
3. McNeil, J.C.; Sommer, L.M.; Boyle, M.; Hogan, P.; Vallejo, J.G.; Hultén, K.G.; Flores, A.R.; Kaplan, S.L.; Fritz, S. Cefazolin Inoculum Effect and Methicillin-Susceptible *Staphylococcus aureus* Osteoarticular Infections in Children. *Antimicrob Agents Chemother* **2020**, *64*, e00703-20, doi:10.1128/AAC.00703-20.
4. Chen, J.-L.; Huang, T.-Y.; Hsu, W.-B.; Lee, C.-W.; Chiang, Y.-C.; Chang, P.-J.; Peng, K.-T. Characterization of Methicillin-Resistant *Staphylococcus aureus* Isolates from Periprosthetic Joint Infections. *Pathogens* **2022**, *11*, 719, doi:10.3390/pathogens11070719.
5. McNeil, J.C.; Vallejo, J.G.; Kok, E.Y.; Sommer, L.M.; Hultén, K.G.; Kaplan, S.L. Clinical and Microbiologic Variables Predictive of Orthopedic Complications Following *Staphylococcus*

aureus Acute Hematogenous Osteoarticular Infections in Children. *Clin Infect Dis* **2019**, *69*, 1955–1961, doi:10.1093/cid/ciz109.

6. Peyrani, P.; Allen, M.; Seligson, D.; Roberts, C.; Chen, A.; Haque, N.; Zervos, M.; Wiemken, T.; Harting, J.; Christensen, D.; et al. Clinical Outcomes of Osteomyelitis Patients Infected with Methicillin-Resistant *Staphylococcus aureus* USA-300 Strains. *Am J Orthop (Belle Mead NJ)* **2012**, *41*, 117–122.
7. Martínez-Aguilar, G.; Avalos-Mishaan, A.; Hulten, K.; Hammerman, W.; Mason, E.O.J.; Kaplan, S.L. Community-Acquired, Methicillin-Resistant and Methicillin-Susceptible *Staphylococcus aureus* Musculoskeletal Infections in Children. *The Pediatric Infectious Disease Journal* **2004**, *23*, 701–706, doi:10.1097/01.inf.0000133044.79130.2a.
8. Valour, F.; Rasigade, J.-P.; Trouillet-Assant, S.; Gagnaire, J.; Bouaziz, A.; Karsenty, J.; Lacour, C.; Bes, M.; Lustig, S.; Bénet, T.; et al. Delta-Toxin Production Deficiency in *Staphylococcus aureus:* A Diagnostic Marker of Bone and Joint Infection Chronicity Linked with Osteoblast Invasion and Biofilm Formation. *Clinical Microbiology and Infection* **2015**, *21*, 568.e1-568.e11, doi:10.1016/j.cmi.2015.01.026.
9. Lin, Y.-T.; Tsai, J.-C.; Yamamoto, T.; Chen, H.-J.; Hung, W.-C.; Hsueh, P.-R.; Teng, L.-J. Emergence of a Small Colony Variant of Vancomycin-Intermediate *Staphylococcus aureus* in a Patient with Septic Arthritis during Long-Term Treatment with Daptomycin. *J Antimicrob Chemother* **2016**, *71*, 1807–1814, doi:10.1093/jac/dkw060.
10. Bouiller, K.; Hocquet, D.; Sauget, M.; Bertrand, X.; Chirouze, C. Epidemiology and Risk Factors of *Staphylococcus aureus* CC398 Bone and Joint Infections. *BMC Infect Dis* **2020**, *20*, 384, doi:10.1186/s12879-020-05098-0.
11. Banerjee, B.; Shaw, T.; Mukhopadhyay, C.; Bhat N, S.; Singh, B.M.K. Epidemiology, Resistance Characteristics, Virulence Determinants, and Treatment Outcomes of *Staphylococcus aureus* Bone and Joint Infections: A One-Year Prospective Study at a Tertiary Care Hospital in India. *Pathog Glob Health* **2020**, *114*, 482–486, doi:10.1080/20477724.2020.1823108.
12. Wildeman, P.; Tevell, S.; Eriksson, C.; Lagos, A.C.; Söderquist, B.; Stenmark, B. Genomic Characterization and Outcome of Prosthetic Joint Infections Caused by *Staphylococcus aureus*. *Sci Rep* **2020**, *10*, 5938, doi:10.1038/s41598-020-62751-z.
13. Cafiso, V.; Lo Verde, F.; Zega, A.; Pigola, G.; Rostagno, R.; Borrè, S.; Stefani, S. Genomic Characterization of a New Biofilm-Forming and Adhesive ST398 Human-Adapted MSSA Lineage Causing Septic Knee Arthritis Following Surgical Reconstruction. *Microorganisms* **2021**, *9*, 305, doi:10.3390/microorganisms9020305.
14. Gaviria-Agudelo, C.; Aroh, C.; Tareen, N.; Wakeland, E.K.; Kim, M.; Copley, L.A. Genomic Heterogeneity of Methicillin Resistant *Staphylococcus aureus* Associated with Variation in Severity of Illness among Children with Acute Hematogenous Osteomyelitis. *PLoS One* **2015**, *10*, e0130415, doi:10.1371/journal.pone.0130415.
15. Trobos, M.; Firdaus, R.; Svensson Malchau, K.; Tillander, J.; Arnellos, D.; Rolfson, O.; Thomsen, P.; Lasa, I. Genomics of *Staphylococcus aureus* and *Staphylococcus epidermidis* from Periprosthetic Joint Infections and Correlation to Clinical Outcome. *Microbiol Spectr* **2022**, *10*, e0218121, doi:10.1128/spectrum.02181-21.
16. Muñoz-Gallego, I.; Viedma, E.; Esteban, J.; Mancheño-Losa, M.; García-Cañete, J.; Blanco-García, A.; Rico, A.; García-Perea, A.; Ruiz Garbajosa, P.; Escudero-Sánchez, R.; et al. Genotypic and Phenotypic Characteristics of *Staphylococcus aureus* Prosthetic Joint Infections: Insight on the Pathogenesis and Prognosis of a Multicenter Prospective Cohort. *Open Forum Infectious Diseases* **2020**, *7*, ofaa344, doi:10.1093/ofid/ofaa344.
17. McCaskill, M.L.; Mason, E.O.J.; Kaplan, S.L.; Hammerman, W.; Lamberth, L.B.; Hultén, K.G. Increase of the USA300 Clone Among Community-Acquired Methicillin-Susceptible *Staphylococcus aureus* Causing Invasive Infections. *The Pediatric Infectious Disease Journal* **2007**, *26*, 1122–1127, doi:10.1097/INF.0b013e31814536e0.
18. Muñoz-Gallego, I.; Lora-Tamayo, J.; Pérez-Montarelo, D.; Brañas, P.; Viedma, E.; Chaves, F. Influence of Molecular Characteristics in the Prognosis of Methicillin-Resistant *Staphylococcus*

aureus Prosthetic Joint Infections: Beyond the Species and the Antibiogram. *Infection* **2017**, *45*, 533–537, doi:10.1007/s15010-017-1011-6.
19. Sdougkos, G.; Chini, V.; Papanastasiou, D.A.; Christodoulou, G.; Tagaris, G.; Dimitracopoulos, G.; Spiliopoulou, I. Methicillin-Resistant *Staphylococcus aureus* Producing Panton-Valentine Leukocidin as a Cause of Acute Osteomyelitis in Children. *Clin Microbiol Infect* **2007**, *13*, 651–654, doi:10.1111/j.1469-0691.2007.01713.x.
20. Valour, F.; Tasse, J.; Trouillet-Assant, S.; Rasigade, J.-P.; Lamy, B.; Chanard, E.; Verhoeven, P.; Decousser, J.-W.; Marchandin, H.; Bès, M.; et al. Methicillin-Susceptible *Staphylococcus aureus* Clonal Complex 398: High Prevalence and Geographical Heterogeneity in Bone and Joint Infection and Nasal Carriage. *Clin Microbiol Infect* **2014**, *20*, O772-775, doi:10.1111/1469-0691.12567.
21. Muñoz-Gallego, I.; Meléndez-Carmona, M.Á.; Lora-Tamayo, J.; Garrido-Allepuz, C.; Chaves, F.; Sebastián, V.; Viedma, E. Microbiological and Molecular Features Associated with Persistent and Relapsing *Staphylococcus aureus* Prosthetic Joint Infection. *Antibiotics (Basel)* **2022**, *11*, 1119, doi:10.3390/antibiotics11081119.
22. Moutaouakkil, K.; Abdellaoui, H.; Arhoune, B.; Atarraf, K.; El Fakir, S.; Yahyaoui, G.; Mahmoud, M.; Afifi, M.A.; Oumokhtar, B. Paediatric Osteoarticular Infections Caused by *Staphylococcus aureus* Producing Panton-Valentine Leucocidin in Morocco: Risk Factors and Clinical Features. *Afr J Paediatr Surg* **2022**, *19*, 78–82, doi:10.4103/ajps.AJPS_18_21.
23. Jiang, B.; Wang, Y.; Feng, Z.; Xu, L.; Tan, L.; Zhao, S.; Gong, Y.; Zhang, C.; Luo, X.; Li, S.; et al. Panton-Valentine Leucocidin (PVL) as a Potential Indicator for Prevalence, Duration, and Severity of *Staphylococcus aureus* Osteomyelitis. *Front Microbiol* **2017**, *8*, 2355, doi:10.3389/fmicb.2017.02355.
24. Bocchini, C.E.; Hulten, K.G.; Mason, E.O.; Gonzalez, B.E.; Hammerman, W.A.; Kaplan, S.L. Panton-Valentine Leukocidin Genes Are Associated with Enhanced Inflammatory Response and Local Disease in Acute Hematogenous *Staphylococcus aureus* Osteomyelitis in Children. *Pediatrics* **2006**, *117*, 433–440, doi:10.1542/peds.2005-0566.
25. Belthur, M.V.; Birchansky, S.B.; Verdugo, A.A.; Mason, E.O.; Hulten, K.G.; Kaplan, S.L.; Smith, E.O.; Phillips, W.A.; Weinberg, J. Pathologic Fractures in Children with Acute *Staphylococcus aureus* Osteomyelitis. *J Bone Joint Surg Am* **2012**, *94*, 34–42, doi:10.2106/JBJS.J.01915.
26. Dohin, B.; Gillet, Y.; Kohler, R.; Lina, G.; Vandenesch, F.; Vanhems, P.; Floret, D.; Etienne, J. Pediatric Bone and Joint Infections Caused by Panton-Valentine Leukocidin-Positive *Staphylococcus aureus. Pediatr Infect Dis J* **2007**, *26*, 1042–1048, doi:10.1097/INF.0b013e318133a85e.
27. Gijón, M.; Bellusci, M.; Petraitiene, B.; Noguera-Julian, A.; Glikman, D.; Saavedra-Lozano, J.; Neth, O.; Daskalaki, M.; Zilinskaite, V.; Kaiser-Labusch, P.; et al. Pediatric Community-Acquired Bone and Joint *Staphylococcus aureus* Infections In Europe: Severe Infections Are Associated to Panton-Valentine Leucocidin Presence. *Pediatr Infect Dis J* **2020**, *39*, e73–e76, doi:10.1097/INF.0000000000002640.
28. Ma, D.; Brothers, K.M.; Maher, P.L.; Phillips, N.J.; Simonetti, D.; William Pasculle, A.; Richardson, A.R.; Cooper, V.S.; Urish, K.L. *Staphylococcus aureus* Genotype Variation among and within Periprosthetic Joint Infections. *Journal of Orthopaedic Research* **2022**, *40*, 420–428, doi:10.1002/jor.25031.
29. Kalinka, J.; Hachmeister, M.; Geraci, J.; Sordelli, D.; Hansen, U.; Niemann, S.; Oetermann, S.; Peters, G.; Löffler, B.; Tuchscherr, L. *Staphylococcus aureus* Isolates from Chronic Osteomyelitis Are Characterized by High Host Cell Invasion and Intracellular Adaptation, but Still Induce Inflammation. *International Journal of Medical Microbiology* **2014**, *304*, 1038–1049, doi:10.1016/j.ijmm.2014.07.013.
30. Bouras, D.; Doudoulakakis, A.; Tsolia, M.; Vaki, I.; Giormezis, N.; Petropoulou, N.; Lebessi, E.; Gennimata, V.; Tsakris, A.; Spiliopoulou, I.; et al. *Staphylococcus aureus* Osteoarticular Infections in Children: An 8-Year Review of Molecular Microbiology, Antibiotic Resistance and Clinical Characteristics. *J Med Microbiol* **2018**, *67*, 1753–1760, doi:10.1099/jmm.0.000859.

31. Loss, G.; Simões, P.M.; Valour, F.; Cortês, M.F.; Gonzaga, L.; Bergot, M.; Trouillet-Assant, S.; Josse, J.; Diot, A.; Ricci, E.; et al. *Staphylococcus aureus* Small Colony Variants (SCVs): News From a Chronic Prosthetic Joint Infection. *Front Cell Infect Microbiol* **2019**, *9*, 363, doi:10.3389/fcimb.2019.00363.
32. Collins, A.; Wakeland, E.K.; Raj, P.; Kim, M.S.; Kim, J.; Tareen, N.G.; Copley, L.A.B. The Impact of *Staphylococcus aureus* Genomic Variation on Clinical Phenotype of Children with Acute Hematogenous Osteomyelitis. *Heliyon* **2018**, *4*, e00674, doi:10.1016/j.heliyon.2018.e00674.
33. Carrillo-Marquez, M.A.; Hulten, K.G.; Hammerman, W.; Mason, E.O.; Kaplan, S.L. USA300 Is the Predominant Genotype Causing *Staphylococcus aureus* Septic Arthritis in Children. *Pediatr Infect Dis J* **2009**, *28*, 1076–1080, doi:10.1097/INF.0b013e3181adbcfe.
34. Víquez-Molina, G.; Aragón-Sánchez, J.; Pérez-Corrales, C.; Murillo-Vargas, C.; López-Valverde, M.E.; Lipsky, B.A. Virulence Factor Genes in *Staphylococcus aureus* Isolated From Diabetic Foot Soft Tissue and Bone Infections. *Int J Low Extrem Wounds* **2018**, *17*, 36–41, doi:10.1177/1534734618764237.
